# Routine viral load monitoring and enhanced adherence counselling at a public ART centre in Mumbai, India

**DOI:** 10.1371/journal.pone.0232576

**Published:** 2020-05-05

**Authors:** Chinmay Laxmeshwar, Shrikala Acharya, Mrinalini Das, Padmaja Keskar, Amar Pazare, Nayana Ingole, Preeti Mehta, Pooja Gori, Homa Mansoor, Stobdan Kalon, Pramila Singh, Taanya Mathur, Gabriella Ferlazzo, Petros Isaakidis

**Affiliations:** 1 Médecins Sans Frontières, Mumbai, India; 2 Mumbai Districts AIDS Control Society, Mumbai, India; 3 KEM Hospital, Mumbai, India; 4 Southern Africa Medical Unit, Médecins Sans Frontières, Cape Town, South Africa; University of Arizona College of Medicine, UNITED STATES

## Abstract

**Background:**

Routine viral-load (VL) measurements along with enhanced adherence counselling (EAC) are recommended to achieve virological suppression among people living with HIV/AIDS (PLHA) on anti-retroviral therapy (ART). The Mumbai Districts AIDS Control Society along with Médecins Sans Frontières has provided routine VL measurements and EAC to PLHA on ART at King Edward Memorial (KEM) hospital, Mumbai since October-2016. This study aims to describe the initial VL results and impact of EAC on viral suppression and factors associated with initial viral non-suppression among patients with an initial detectable VL, in a cohort of patients tested between October-2016 and September-2018.

**Methods:**

This is a descriptive study of PLHA on ART who received VL testing and EAC during October-2016 to September-2018. Log-binomial regression was used to identify factors associated with a high VL.

**Results:**

Among 3849 PLHA who underwent VL testing, 1603(42%) were female and median age was 42 years (IQR:35–48). Majority were referred for routine testing (3432(89%)) and clinical/immunological failure (233(6%)). Overall, 3402(88%) PLHA had suppressed VL at initial testing. Among 3432 tested for routine monitoring, 3141(92%) had VL suppressed. Of 291 with VL>1000c/ml, 253(87%) received EAC and after repeat VL, 70(28%) had VL<1000c/ml. Among 233 referred for clinical/immunological failure, 122(52%) had VL>1000c/ml and 109 have been switched to second-line ART.

CD4 count<500 (aOR-5.0[95%CI 3.8–6.5]), on ART for<5 years (aOR-1.5[1.1–2.0]) and age<15 years (aOR-5.2[3.0–8.9]) were associated with an initial VL>1000c/ml. Factors associated with follow-up VL suppression included EAC (p<0.05) and being on second-line ART (p<0.05).

**Conclusion:**

Results from a routine VL program in public sector in India were encouraging and in line with UNAIDS 90-90-90 targets. Routine VL monitoring along with EAC resulted in early switch to alternative optimised regimens while also preventing unnecessary switches. Along with the vital scale up of routine VL monitoring, implementation of enhanced adherence strategies for patients with detectable viral load should be ensured.

## Introduction

The HIV/AIDS epidemic continues with 36.9 million people living with HIV/AIDS (PLHA) globally in 2017 [1]. India, with an estimated 2.1 million PLHA had the third highest number of PLHA in the world in 2015 [2]. Coverage of antiretroviral therapy (ART) is increasing with 21.7 million PLHA worldwide on ART in 2017 [1]. In India, about 1.1 million PLHA were estimated to be on ART in 2015 [3]. India is committed to achieve the UNAIDS 90-90-90 targets, which specify that 90% of PLHA should know their HIV status, 90% diagnosed with HIV should be on ART and 90% of those on ART should have viral suppression [3,4].

To achieve this viral suppression, routine HIV viral load (VL) monitoring is necessary. The World Health Organization (WHO) recommends VL as the preferred approach to ART monitoring compared to clinical or immunological criteria [5]. This is due to the fact that a decrease in CD4 count, which is a marker of immunological failure, occurs as result of viral replication, which can thus be considered as an endpoint [6]. Routine VL has been shown to lead to earlier detection of treatment failure compared to immunological and clinical criteria. This can lead to prompt and correct switches to optimised second or third line ART, which in turn can improve treatment outcomes [7,8]. Routine VL testing can also help prevent unnecessary switches to second-line or third-line ART [9].

From a drug-resistance perspective, early detection of unsuppressed viral load and treatment failure through routine VL measurement can contribute to prevent accumulation of thymidine analogue mutations, which have the potential to reduce the effectiveness of second-line ART regimens [9–12]. Development of HIV drug- resistance is a serious issue for HIV control programs and can wipe out the gains made so far [13,14]. This would also lead to increased costs for HIV programs, which would be a high burden for low resource settings [8,12]. Mathematical modelling has shown that use of routine VL can also lead to reduced HIV transmission [15].

Along with identifying failures, routine VL has also been shown to help with adherence [9,16–18]. It has been postulated that just being informed about a high viral load has positive effect on adherence of a patient [19]. Adherence counselling encourages patients to discuss barriers to treatment and develop a plan accordingly with the counsellor. WHO recommends adherence support between two VL measurements in order to define failure [20]. Use of routine VL coupled with enhanced adherence counselling has shown to lead to optimal utilisation of ART services, with improved outcomes among patients [19].

Routine VL testing in India is was launched in 2018 [3]. The National AIDS Control Organisation (NACO) has recommended in 2018 the use of routine VL monitoring with the aim to identify patients with early treatment failure and those in need switch to optimised ART regimen. Adherence support has also been planned by providing three counselling sessions to patients with detectable VL, provision of a case manager and if necessary daily supervised ingestion of treatment [3]. Prior to the introduction of these national guidelines, in 2016, Mumbai Districts AIDS Control Society (MDACS) and the international humanitarian non-governmental organisation Médecins Sans Frontières (MSF) started providing routine viral load measurements and enhanced adherence counselling to a cohort of patients taking ART at King Edward Memorial (KEM) hospital in Mumbai. This study was conducted with an aim to describe the initial viral load, the impact of enhanced adherence counselling on VL suppression for patients with VL >1000c/ml and the factors associated with initial viral non-suppression among PLHA tested between October 2016 and September 2018.

## Methods

### Study design

This is a descriptive study using routinely collected programme data.

### Setting

Mumbai is a megacity in Maharashtra, India with a population of about 12.5 million people [21]. The city is home to 25% of PLHA in the state. The Mumbai Districts AIDS Control Society is responsible for implementation of the National AIDS Control Program in the city. In 2015, there were 75,220 PLHA registered with MDACS while 62% among them were on active care [22]. ART is provided through 14 ART centres spread across the city. First- and second-line ART are available at all centres while third-line ART is available at the Centre of Excellence at Sir J.J. group of hospitals.

The ART centre at King Edward Memorial (KEM) hospital is a high burden centre providing services to about 4200 PLHA. The centre provides first- and second line ART. All regimens are designed as per national guidelines [23]. The centre is manned by doctors, nurses, counsellors, peer educators and pharmacist. Clinical decision making is supported by a District AIDS Clinical Expert Panel (DACEP). Any changes to ART regimens can be made only by this panel.

Since October 2016, MDACS along with MSF has been providing VL monitoring along with enhanced adherence counselling for PLHA on ART at KEM hospital. PLHA on ART for more than 6 months or those with signs of clinical failure were eligible for VL measurement. In case the VL was more than 1000 c/ml, then the PLHA was referred to a counsellor for enhanced adherence counselling spread over three months before VL was retested. It was recommended that at least one EAC session be conducted with the patient upon receiving a high viral load result and before retesting. The first session would be offered the same day the viral load result was received. The second session was offered one month later and before the 2nd viral load. More sessions could be offered, according to the patient’s needs.

Enhanced adherence counselling followed the motivation-information-behaviour skill model. During the first session, patients with high viral load were invited during the counselling sessions to assess the barriers they face in adherence to their treatment. The counsellor helps the patient assess the information and motivation level of the patient and serves as a facilitator to fill the relevant gaps. In addition to providing information about HIV and viral load, providing emotional support is a very important component of EAC, as many patients report facing stigma, feeling depressed/or suffering from other mental health problems. Keeping all this in mind, an individualized adherence plan is developed which further enables the patient to take their treatment successfully. The second EAC session focuses on reviewing the plan with the patient and supporting them, where required. The end goal for EAC is to ensure that patients take their medication timely and in an effective manner.

Post EAC, the VL was retested, three months after the first measurement. In case the VL result was still more than 1000c/ml, then the patient was referred to the District AIDS Clinical Expert Panel (DACEP) in KEM hospital. The panel would decide on the appropriate ART regimen based on national guidelines. Patients may be referred directly to DACEP in case of clinical/immunological failure. Patients referred for presumptive clinical/immunological failure also underwent viral load testing. However, their regimen could be changed by DACEP even before EAC and repeat VL testing, based on clinical criteria under the national guideline.

For all patients with supressed VL, national guidelines now recommend routine VL monitoring, to be repeated once a year. Standard WHO definitions were used for defining clinical, immunological and virological failure (Table 1) [24].

**Table 1:** WHO definitions of clinical, immunological and virological failures.

Type of failure	Definition
Clinical failure	Adults and adolescents–New or recurrent clinical event indicating severe immunodeficiency (WHO clinical stage 4 condition) after 6 months of effective treatment.
	Children–New or recurrent clinical event indicating advanced or severe immunodeficiency (WHO clinical stage 3 and 4 clinical condition with the exception of TB) after 6 months of effective treatment.
Immunological failure	Adults and adolescents–CD4 count at or below 250 cells/mm^3^ following clinical failure OR persistent CD4 levels below 100 cells/mm^3^.
	Children<5 years–Persistent CD4 levels below 200 cells/mm^3^.
	Children>5 years–Persistent CD4 levels below 100 cells/mm^3^.
Virological failure	Among patients on ART for more than six months, virological failure is viral load above 1000 copies/ml based on two consecutive viral load measurements in 3 months, with adherence support following the first viral load test.

VL were done using Xpert HIV-1 Viral Load (Cephid) platform.

### Study population

All PLHA tested for HIV-VL, irrespective of the reason for testing (routine, clinical/immunological failure and other reasons), between October 2016 and September 2018 were included in this study.

### Data collection and variables

Details of all patients who underwent VL testing were captured by a nurse in patient cards at the ART centre. The data then was entered into a Microsoft Excel 2010 database by a dedicated data encoder. Socio-demographic variables, ART regimen and CD4 counts were captured from patient cards. VL result was entered from lab reports. Dates of EAC were provided by the counsellor to the data encoder and were entered in a Microsoft Excel 2010 database. A cut-off date of 31 December 2018 was used for follow-up viral load and regimen switches.

### Data analysis

Data was imported into SPSS (Version 20, 2011; IBM Inc., Chicago, IL, USA) for analysis. Categorical variables among the socio-demographic characteristics of the study participants were described using numbers and proportions, and continuous variables described using median and inter quartile range (IQR). Bivariate analysis was conducted to study the relationships between demographic and select variables with VL>1000c/ml. Those with a p-value of less than 0.2 were included in the multivariate model along with factors considered clinically and epidemiologically significant. Log binomial regression was used to identify factors associated with VL>1000c/ml. A p-value of 0.05 or below was considered statistically significant.

### Ethics

Approval was obtained from the Mumbai Districts AIDS Control Society for conducting this study (Reference number MDACS/10383/PD dated 15^th^ February 2018). This research also fulfilled the exemption criteria set by the Médecins Sans Frontières Ethics Review Board (Geneva, Switzerland) for a posteriori analyses of routinely collected clinical data and thus did not require MSF ERB review. It was conducted with permission from Medical Director, Operational Centre Brussels, Médecins Sans Frontières. All data was annonymised before it was obtained for analysis.

## Results

A total of 3849 PLHA were tested for HIV viral load during the study period. Median age was 42 years (IQR– 35–48 years) and 42% were female. Socio-demographic and clinical characteristics of the patients are described in Table 2.

**Table 2:** Socio-demographic and clinical characteristics of PLHA tested for HIV viral load at ART centre, KEM hospital, Mumbai during October 2016 –September 2018.

	n (%)
Number tested for HIV-VL*	3849
Median Age (IQR^#^)	42 (35–48)
Sex	
Male	2246 (58)
Female	1603 (42)
ART^$^ regimen	
First line ART	3648 (95)
Alternative first line ART	76 (2)
Second line ART	125 (3)
Reason for referral	
Routine testing	3432 (89)
Clinical/immunological failure	233 (6)
Other	184 (5)
Median CD4 (IQR) (n = 3811)	517 (360–699)
Median time on ART in months (IQR) (n = 3832)	40 (23–60)

* VL–Viral load

^#^ IQR–Inter quartile range

^$^ ART–Anti retroviral therapy

### HIV viral load cascade

**Fig 1:**
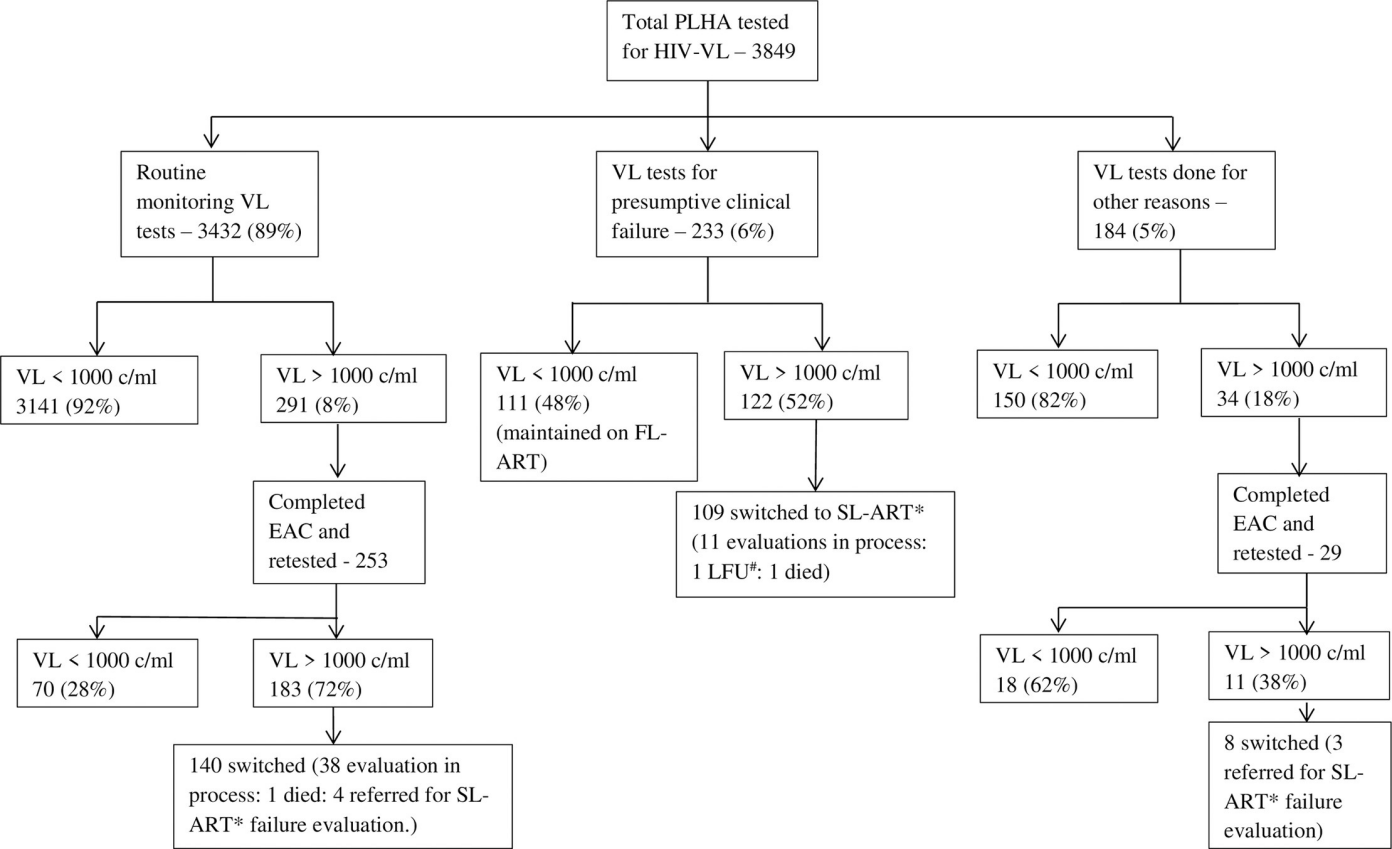
HIV viral-load testing cascade among people living with HIV/AIDS on ART at KEM hospital, Mumbai, India. * Second Line Anti-retroviral therapy: # Lost to follow up.

Among all the viral load tests, 3432 (89%) were done for routine monitoring, 233 (6%) were done for presumptive clinical/immunological failure, while 184 (5%) were done for other reasons. The viral load testing cascade has been described in Fig 1. Overall, 3402 (88%) of patients tested were found to have a VL<1000c/ml. Among those tested for routine monitoring, 92% (3141/3432) had VL less than 1000c/ml, while among those tested because of clinical/immunological failure, 48% (111/233) had a VL<1000c/ml. Those with clinical/immunological failure and VL<1000c/ml were maintained on the same regimen while adherence was reinforced by use of EAC. Among the 291 tested for routine VL testing who had VL>1000c/ml, 253 (87%) attended at least one EAC during the study period and underwent VL retesting after at least 3 months. Among 253 who underwent EAC, 20 (8%) underwent one EAC session, 123 (48%) two sessions and 111 (44%) three or more sessions. After EAC, 70 (28%) had their VL suppressed and were kept on the same ART regimen. The remaining 183 (72%) were declared treatment failure and referred for ART switch. Among these, 140 (77%) were effectively switched during the study period.

### Factors associated with initial HIV viral load

In bivariate analysis, age (p <0.001), time on the ART (p<0.001), CD4 count (p<0.001) and reason for referral (p<0.001) were significantly associated with high initial viral load. Sex (p = 0.179) was not seen to be significantly associated with initial VL.

In multivariate analysis (Table 3), age <15 years, less than 5 years on ART, CD4 count less than 500 and being referred for clinical/immunological failure were seen to be associated with baseline VL>1000c/ml.

**Table 3:** Factors affecting initial VL among patients on ART at KEM hospital, Mumbai.

Variable		Viral load			Risk ratio (95% CI)	
		>1000c/ml n (%)	<1000 c/ml n (%)	Total		
Age						
	< = 15 years	27 (25)	79 (75)	106	5.16 (2.99–8.88)*	
	16–45 years	290 (12)	2159 (88)	2449	1.29 (1.01–1.66)*	
	>45 years	130 (10)	1164 (90)	1294	1	
Sex						
	Male	274 (12)	1972 (88)	2246	1	
	Female	173 (11)	1430 (89)	1603	1.03 (0.82–1.31)	
Reason for referral						
	Routine	291 (8)	3141 (92)	3432	1	
	Clinical/immunological failure	122 (52)	111 (48)	233	8.13 (6.03–10.96)*	
	Other	34 (18)	150 (82)	184	1.62 (1.01–2.59)*	
Duration on ART						
	>5 years	74 (8)	875 (92)	949	1	
	<5 years	363 (13)	2520 (87)	2883	1.47 (1.1–1.98)*	
CD4 count (cells/mm^3^)						
	>500	81 (4)	1923 (96)	2004	1	
	<500	359 (20)	1448 (80)	1807	5.01 (3.82–6.56)*	

* - p < 0.05

### Factors associated with post EAC viral load suppression among those tested for routine testing

Among those referred for routine VL testing and who had a baseline VL of more than 1000c/ml, post EAC, 253 had a repeated VL testing after 3 months (Fig 1). Number of EAC sessions was seen to have a statistically significant association with VL suppression (p<0.05). Among those who received only 1 EAC, 10 (50%) had their VL supressed on repeat testing. While for those who had received more than 1 EAC only 60 (26%) had their VL supressed. Also, patients on second-line ART were seen to have significantly (p = 0.015) better suppression (7, 58%) compared to those on first-line ART (63, 26%).

## Discussion

The study shows a virological suppression of 88% in the KEM hospital cohort at the beginning of the intervention, while a suppression of 92% was observed among the subgroup of patients tested for routine monitoring. These are very encouraging findings and in line with the target of 90% virological suppression set by UNAIDS. Among patients tested for routine monitoring and who had VL>1000c/ml, 28% had a follow-up VL<1000c/ml and their ART regimen was maintained, while the rest were referred for switch to second-line ART regimen. Almost 77% of those referred were switched during the study period. Among those tested for clinical/immunological failure, almost half had VL<1000c/ml and their regimens were maintained, as per national guidelines, thus preventing unnecessary switches. The role of routine viral load in earlier identification of failures and, earlier switches to second-line ART and improved treatment outcomes, while at the same time preventing unnecessary switches has been reported by many studies around the world [6–14,16–19,25–31].

Multivariate analysis revealed that age less than 15 years was significantly associated with a higher VL. Many studies around the world have reported lower age as a risk factor for higher VL [16,32,33]. Maintaining adherence among adolescents and children is a well-recognised challenge [34]. Viral suppression has been reported to be low in these age groups even post counselling [35]. Hence, it is important that special attention is given to these age groups. Context specific and age specific interventions might help improve adherence and thus response to treatment in this vulnerable group. Immunological status, evidenced by CD4 count was also seen to be associated with VL, with PLHA with CD4 less than 500 showing more virological failure than those with CD4 count more than 500. This result is in concordance with multiple studies reporting similar findings [10,19,36].

Among those with baseline VL>1000c/ml, follow-up viral suppression was significantly associated with EAC. Many studies around the world have proved effectiveness of EAC in helping patients adhere to their treatment and thus leading to a decreased VL. A study from Swaziland had reported 61% suppression post-EAC [19], while another study from South Africa reports a 41% post-EAC suppression [10]. In our study, looking at the number of EAC sessions and its effect on VL suppression, it was observed that those who had only one session showed better suppression than those who had more than one. This might be because only those who had a higher degree or long-standing adherence issues might have been selected for more than one session. Previous studies have reported that it is the support rather than the number of sessions which lead to improved adherence [19]. Thus, while introducing routine viral load, programs should also pay enough attention on adherence counselling and support. Follow-up viral suppression was also higher in those on second-line ART, which follows a study done in Uganda [33].

The strengths of this study include that it reports a large cohort of PLHA on ART at a high burden public ART centre. The participants in this study received access to routine VL before the release and implementation of national guidelines, and thus represent one of the earliest cohorts in the country to receive access to routine VL. While patients received VL testing, they were also adequately supported to improve their adherence by a trained counsellor. However, the follow-up period for the patients was short, which is a limitation of this study. Providing routine VL and EAC over a longer period should inform about the long term efficacy and effectiveness of EAC in the population.

In conclusion, viral suppression among PLHA on ART was in line with the UNAIDS 90-90-90 targets in the study cohort. The use of routine viral load led to early identification of treatment failures and referrals to second- and third-line regimens, while also preventing unnecessary switches. Enhanced adherence counselling played a significant role in viral load suppression among those identified with a high initial viral load. While scaling up the essential access to viral load monitoring and ensuring rapid switch to second- and third-line ART regimens for failing patients, national HIV programmes should put efforts in the integration of adherence support strategies for the best results and adequate resource utilisation.
